# Tinnitus in elderly population: clinic correlations and impact upon QoL

**Published:** 2011-11-24

**Authors:** A. Negrila-Mezei, R. Enache, C. Sarafoleanu

**Affiliations:** ENT&HNS Department, “Sfanta Maria” Hospital, Bucharest Romania

**Keywords:** tinnitus, cooping strategies, elderly

## Abstract

**Introduction:** Tinnitus is a common health problem that affects between 10 - 30% of the population, approximately 3 - 4% presenting to the doctor at least once in their life. There are many causes that lead to tinnitus in elderly population, including otology, metabolic, neurologic or cardiovascular conditions. The aim of this study was to determine the association of tinnitus with these chronic comorbidities among elderly community and its impact upon their quality of life.

**Materials and Methods:** We performed a clinical retrospective study on 471 ENT patients hospitalized for various diseases, up to 60 years old, for a period of 24 months. All subjects were assessed for subjective tinnitus, neuro-vascular comorbidities and QoL by use of the brief version of the World Health Organization QoL instrument.

**Results: **Tinnitus was reported in 114 patients, giving a prevalence of 24,2%. Variables like gender, residence, economic status, alcohol or smoking were not significantly associated with tinnitus. On the other hand, otic and sinonasal pathology, dizziness, hypertension, arteriosclerosis or diabetes were significantly correlated. All patients with tinnitus presented a more negative perception of their overall health and a poorer QoL, compared to those without.

**Conclusions: **Tinnitus is a common pathology among elderly community. Its association with chronic treatable health comorbidities reduces QoL and highlights the need of cooping strategies among this group population.

**Abbreviations:** RR = Relative Risk; 95%CL=95%Confidence Level; OR = Ods Ratio; QoL= quality of life

## Introduction

Tinnitus represents one of the most common and distressing otology problems, which affects between 10 - 30% of the population, approximately 3 - 4% presenting to the doctor at least once in their life.

**Figure 1:**
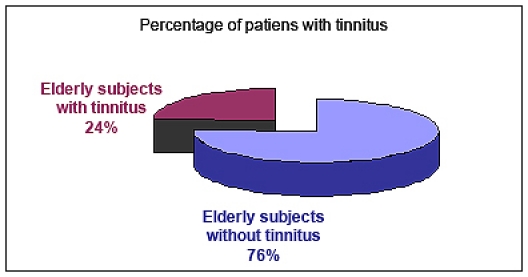
Percentage of patients with tinnitus

It is one of the most important symptoms in neurootology besides the hearing loss, vertigo, dizziness and nausea [**1**] and it can cause various somatic and psychological disorders that interfere with the quality of life [**2**].

There are many causes which can lead to tinnitus, like cardio-vascular pathologies, otic pathologies, head injury, electric shock, otic barotraumas, and as a side effect of many drugs. In addition, its effect on the functioning of affected persons makes it a significant contributor to morbidity in the elderly [**3**],[**4**].

Tinnitus can be classified into subjective tinnitus, in which the sound originates within the ear or head, and objective tinnitus, in which patients hear real sounds. The objective tinnitus may be pulsatile sounds caused by vibrations from turbulent blood flow that reaches the cochlea or clicking or low-pitched buzzing indicative of palatal myoclonus or contractions of the tensor tympani or stapedius muscle [**5**].

There are many causes leading to tinnitus in elderly population, including otology, metabolic, neurologic or cardiovascular conditions. The aim of this study was to determine the association of subjective tinnitus with these chronic comorbidities among elderly community and its impact upon their quality of life.

## Materials and Methods

We performed a clinical retrospective study on 471 ENT patients hospitalized for various diseases, up to 60 years old, for a period of 24 months. All the subjects were assessed for subjective tinnitus, neuro-vascular comorbidities and QoL .

The diagnosis protocol consisted in face-to-face interviews, clinical examination, audiological evaluation and imaging investigations.

In order to evaluate the patient’s QoL, all of them completed the brief version of the World Health Organization QoL instrument (WHOQoL-Bref) [**6**].

Study inclusion criteria: 1. The patients should have problems with their tinnitus (for example tinnitus is audible in many acoustic environments, disturbs sleep, or is a dominating problem that affects the quality of life) 2. Occurrence and persistence of tinnitus at least 6 months.

Exclusion criteria: 1. Patients with tinnitus, due to acute conditions, such acute otitis or earwax 2. Persons with difficulty in performing audiometry (cognitive deficits or inconsistent responses) 3. Acoustic neurinoma

The data analysis was made by using a SAS software suite. Because most of our variables are dichotomous, except the age and the QoL scores, we used nonparametric techniques in univariate analysis. The clinical correlations were explored with logistic regression analysis.

## Results

**Figure 2:**
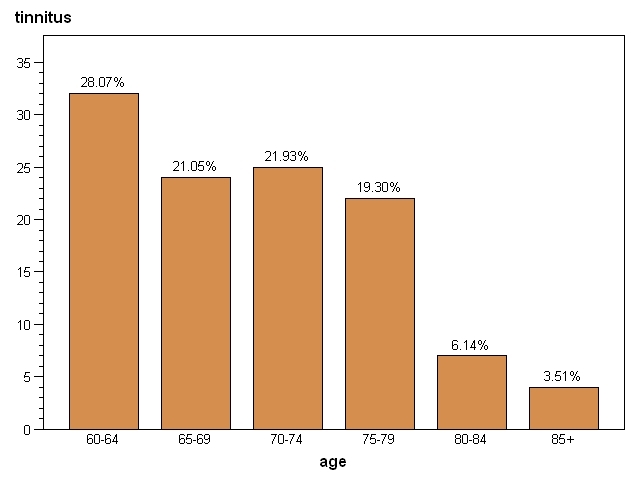
Percentage distribution of tinnitus patients according to age

Our study group consisted of 272 (57.7%) male and 199 (42.3%) female subjects, with a mean age of 70. Tinnitus was reported in 114 patients giving a prevalence of 24.2%. The age distribution presented in (**Figure 2**) shows that the difference in the rates of tinnitus between the age groups was not statistically significant. Despite that, there is a significant difference between young elderly (lt 70 years old, 49.12%) and the older (gt 75 years, 28.95%).

**Figure 3:**
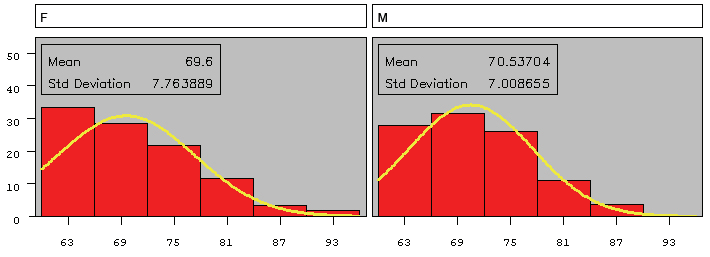
Age distribution of tinnitus patients according to sex

Regarding the age distribution of tinnitus patients according to sex, the Kolmogorov-Smirnov Goodness-of-Fit Tests for Normal Distribution, is D=0.108 (p=0.081) regarding the women and D=0.093 (p>0.150) assigned to men. At the level of significance alpha=0.05, the decision is to accept the null hypothesis that the samples follow a normal distribution (**Fig. 3**).

Levene's Test for Homogeneity of age variance according to sex shows that the variances are equal (p=0.4277).

The CV (Coefficient Variation) is 11.5 respectively 9.93. The difference between the two means it is not significant (as seen in **Table 1**).

**Table 1:** Test Differences Between Means Adjustment for Multiple Comparisons: Tukey-Kramer

sex	age MEAN	H0: Mean f = Mean m
		Pr > |t|
f	69.6	0.5020
m	70.53	

The Chi-square test calculates approximate P values, and the Yates' continuity correction is designed to make the approximation better. Without the Yates' correction, the P values are too low. We did not use the exact Fisher test because the number of observations obtained for analysis was not small for only one degree of freedom. The univariate analysis revealed that, at the 0.05 level of significance, the variables sex, otology conditions, sinonasal conditions, dizziness, hypertension and arteriosclerosis proved to be significantly associated with tinnitus. The same analysis showed that smoking and alcohol consumption variables were not significantly associated with tinnitus (**Table 2**).

**Table 2:** Test of association with Tinnitus

Clinical	Chi-Square	Prob	Chi-square with Yates' correction	Prob
Sex	6.6429	0.0100	6.0935	0.0136
Otology conditions	31.0355	<.0001	29.6678	<.0001
Sinonasal conditions	46.5405	<.0001	45.0341	<.0001
Dizziness	8.0714	0.0045	7.4006	0.0065
Hypertension	30.2872	<.0001	29.0971	<.0001
Arteriosclerosis	19.0602	<.0001	18.1270	<.0001
Alcohol consumption	2.0257	0.1547	1.6830	0.1945
Smoking	0.1199	0.7292	0.0473	0.8279

Analyzing the risk factors for tinnitus, only the exposure variables such as sex, otology condition, dizziness, hypertension and arteriosclerosis were found to be a risk factor for tinnitus (**Table 3**). 

**Table 3:** Risk factor for Tinnitus

Variables	RR	95% CL	Odds Ratio	Asymptotic 95% CL	Exact 95% CL
Sex	1.5187	1.1040-2.0893	1.7426	1.1395-2.6648	1.1139-2.7261
Otology conditions	2.4217	1.7856-3.2845	3.5039	2.2234-5.5219	2.1616-5.6532
Sinonasal conditions	0.3321	0.2378-0.4636	0.2247	0.1438-0.3510	0.1400-0.3592
Dizziness	1.6056	1.1659-2.2113	1.9085	1.2172-2.9923	1.1818-3.0574
Hypertension	2.9545	1.9268-4.5305	3.9318	2.3625-6.5434	2.3182-6.8670
Arteriosclerosis	2.0522	1.4719-2.8611	2.5896	1.6772-3.9984	1.6415-4.1045
RR= Relative Risk, 95%CL=95% Confidence Level

What should be noticed is that OR> RR>1 and 1 is not contained in the 95% CL (Confidence Level). In case of sinonasal conditions, if OR<RR<1 and 95%CL do not contain 1, then this does not represent a risk factor for tinnitus. The correlation matrix shows a low correlation between sex and otology conditions. In the same time, arteriosclerosis is uncorrelated with sex and otology conditions.

We introduced the logistic model tinnitus = sex, otology conditions, arteriosclerosis, with backward selection.

As sex was removed and only the report of otology conditions and of arteriosclerosis remained significantly associated with tinnitus (**Table 4**). 

**Table 4:** Logistic Regression / Partial Output

Analysis of Maximum Likelihood Estimates
Parameter		DF	Estimate	Standard Error	Wald Chi-Square	Pr > ChiSq
Intercept		1	-0.8790	0.1200	53.6817	<.0001
otology conditions	yes	1	0.7046	0.1225	33.0849	<.0001
arteriosclerosis	yes	1	0.5586	0.1179	22.4415	<.0001

Let physical, psychological, social and environmental domain, the raw scores regarding QoL. The coefficient of variation is CV<30 for all domains, so the means are representative for the samples. Wilcoxon Scores (Rank Sums) for variable domains classified by tinnitus, show a difference between the subgroups means, hence, we will conclude that tinnitus affects the quality of life.

**Table 5:** Domain Score Mean: Shapiro-Wilk test for Normality

QoL*	Presence of tinnitus	Score Mean	95% CL for Mean *	Std. Deviation	W Statistic	p Value
Physical domanin	yes	13.37	12.71 - 14.03	3.54	0.94887	0.0003
	no	20.25	19.86 - 20.64	3.76	0.935456	<0.0001
Psichological domanin	yes	13.06	12.45 - 13.67	3.27	0.947856	0.0002
	no	18.21	17.85 - 18.56	3.43	0.93994	<0.0001
Social domanin	yes	6.27	5.95 - 6.60	1.76	0.901858	<0.0001
	no	9.63	9.46 - 9.80	1.64	0.911978	<0.0001
Environmental domanin	yes	16.47	15.66 - 17.29	4.39	0.95629	0.0009
	no	24.31	23.93 - 24.80	4.63	0.943896	<0.0001
*QoL (quality of life), 95%CL – 95% confidence interval QoL was measured with World Health Organization Brief QoL instrument

## Discussions

The World Health Organization defines the elderly in developing countries as persons aged 60 years or over and in developed countries as persons aged 65 years or over [**7**].

Among the morbidities of the elderly, tinnitus is clinically relevant, since it is a symptom rather than a disease, it can affect individual functioning as a whole.

Tinnitus may be caused by numerous otology, metabolic, neurological, orthopedic, cardiovascular, pharmacological, dental and psychological conditions, more than one of which may be present in the same individual [**8**],[**9**].

Sataloff et all [**10**] reported that about 85% of the patients visiting an otologist have tinnitus. Benevides [**11**] reported that tinnitus often accompanies presbyacusis and may be even more troublesome than deafness.

In 2004, a study in Fortaleza, on 260 elderly patients with auditory complaints, revealed that tinnitus was the most prevalent complaint (58.08%), which confirms its frequency in elderly patients seeking otorhinolaryngological help [**12**]. We found a significant difference between the prevalence of tinnitus among young elderly (60-70 years old) subjects (49.12%) and the older (>75 years) group (28.95%).

The main findings in our study were that alcohol consumption and smoking were not associated with the occurrence of tinnitus. The presence of tinnitus is associated with considerable impairment of independent role functioning and with decrement in QoL.

We found a significant difference between the prevalence of tinnitus among young elderly (60-70 years old) subjects (49.12%) and the older (>75 years) group (28.95%).

The involvement of arterial hypertension in the genesis of tinnitus remains controversial. Brohen analyzed a group of hypertensive patients and found tinnitus in 36.0% [**13**]. In 2004, Baraldi correlated tinnitus with hearing loss and found that 34.2% of these patients had high blood pressure [**14**]. In our sample, 276 (58.6%) of the elderly patients were hypertensive, of whom, 184 (52%) had tinnitus. So, our assessment revealed a high correlation between tinnitus and arterial hypertension (p<0.0001).

We observed a significant association between tinnitus and the presence of otology conditions (p<0.0001) and the presence of dizziness (p=0.0065).

The impact of tinnitus upon one person’s QoL can be important having many negative repercussions. Sleep disturbance, defectuous concentration on daily and professional activities, isolation and a poor emotional balance can be often found in tinnitus patients. Anxiety and depression may ensue [**11**],[**12**],[**15-17**].

The statistical results of our study sustain this theory.

## Conclusions

Tinnitus causes much dissatisfaction in elderly patients, since this symptom affects their daily activities and may alter sleeping patterns and the emotional status.

In this study we observed that variables like alcohol or smoking were not significantly associated with tinnitus and otic, sinonasal pathology, dizziness, hypertension or arteriosclerosis as significant correlates.

Its association with chronic treatable health comorbidities reduces QoL and highlights the need of cooping strategies among this group population.
